# Immunity to HIV-1 Is Influenced by Continued Natural Exposure to Exogenous Virus

**DOI:** 10.1371/journal.ppat.1000185

**Published:** 2008-10-24

**Authors:** Christian B. Willberg, J. Jeff McConnell, Emily M. Eriksson, Larry A. Bragg, Vanessa A. York, Teri J. Liegler, Fredrick M. Hecht, Robert M. Grant, Douglas F. Nixon

**Affiliations:** 1 Division of Experimental Medicine, Department of Medicine, University of California San Francisco, San Francisco, California, United States of America; 2 Gladstone Institute of Virology and Immunology, University of California San Francisco, San Francisco, California, United States of America; 3 Division of HIV/AIDS, Department of Medicine, University of California San Francisco, San Francisco, California, United States of America; 4 Positive Health Program, San Francisco General Hospital, University of California San Francisco, San Francisco, California, United States of America; University of Pennsylvania School of Medicine, United States of America

## Abstract

Unprotected sexual intercourse between individuals who are both infected with HIV-1 can lead to exposure to their partner's virus, and potentially to super-infection. However, the immunological consequences of continued exposure to HIV-1 by individuals already infected, has to our knowledge never been reported. We measured T cell responses in 49 HIV-1 infected individuals who were on antiretroviral therapy with suppressed viral loads. All the individuals were in a long-term sexual partnership with another HIV-1 infected individual, who was either also on HAART and suppressing their viral loads, or viremic (>9000 copies/ml). T cell responses to HIV-1 epitopes were measured directly *ex-vivo* by the IFN-γ enzyme linked immuno-spot assay and by cytokine flow cytometry. Sexual exposure data was generated from questionnaires given to both individuals within each partnership. Individuals who continued to have regular sexual contact with a HIV-1 infected viremic partner had significantly higher frequencies of HIV-1-specific T cell responses, compared to individuals with aviremic partners. Strikingly, the magnitude of the HIV-1-specific T cell response correlated strongly with the level and route of exposure. Responses consisted of both CD4^+^ and CD8^+^ T cell subsets. Longitudinally, decreases in exposure were mirrored by a lower T cell response. However, no evidence for systemic super-infection was found in any of the individuals. Continued sexual exposure to exogenous HIV-1 was associated with increased HIV-1-specific T cell responses, in the absence of systemic super-infection, and correlated with the level and type of exposure.

## Author Summary

Serosorting, the practice of seeking to engage in unprotected sexual activities only with partners who are of the same HIV-1 status, is a growing trend. Unprotected sexual intercourse between two HIV-1 infected individuals can lead to consequences such as HIV-1 super-infection. However, continued exposure to HIV-1 may also have an important influence on the immune response. Here, we explored this influence in a cohort of HIV-1 infected individuals who were in long-term partnerships with other HIV-1 infected individuals. We found that individuals, who regularly engaged in unprotected receptive sexual intercourse with an HIV-1 infected viremic partner, displayed higher T cell responses to HIV proteins compared to those who were not regularly exposed to a viremic partner. None of the individuals within this study showed evidence of systemic super-infection. Exposure had limited impact on general activation or poly-functionality. These results are clearly of importance for HIV-1 infected individuals who chose to engage in unprotected sexual activity with other HIV-1 infected individuals. These data also reveal a more general mechanism that occurs in infectious diseases: immune responses to chronic viruses are influenced not only by the virus within the host, but also by exposure to the virus from without.

## Introduction

Immune responses seen during chronic viral infections are thought to be driven only by ‘endogenous’ virus. However, continued exposure to ‘exogenous’ virus could boost anti-viral immunity. HIV-1 infection provides a model to test this hypothesis. Continued sexual intercourse between two HIV-1 infected individuals leads to exposure to exogenous HIV-1. Recent reports have shown that there is a growing trend for serosorting, the practice of seeking to only engage in unprotected sexual activities with partners who are of the same HIV-1 status [1],[2]. This provides a model system in which to investigate the influence of exogenous versus endogenous viral exposure on immunity to a chronic virus infection. Moreover, given the potential risks of super-infection upon re-exposure to HIV-1, understanding the immune responses involved is particularly important [3],[4].

CD8^+^ T cells are thought to play an important role in controlling HIV-1. Model viral infections, such as lymphocytic choriomeningitis (LCMV) and simian immunodeficiency virus (SIV), have shown that the CD8^+^ T cell response is a crucial component in the control or elimination of viral infections [5],[6],[7]. Moreover, the power of CD8^+^ T cell responses have been elegantly shown to be a driving force in the selection of escape variants in SIV [8].

The immune response towards HIV-1 is complex; we set out to determine if T cell mediated responses in an HIV-1 infected individual can be stimulated through exposure to exogenous virus. However, even this simple question has its complexities, as responses to exogenous virus are indistinguishable from responses directed toward the primary infecting virus. To address this we selected individuals with suppressed viral loads while on antiretroviral therapy. Previous studies have shown that viral suppression by highly active antiretroviral therapy (HAART) to below 50 copies/ml leads to the subsequent waning of anti-HIV-1 T cell responses, due to the reduction in viral antigen [9],[10],[11]. Therefore, we hypothesized that individuals with suppressed viral loads while on HAART, who are regularly exposed to an HIV-1 viremic partner, would have greater anti-HIV-1 specific T cell responses compared to individuals who are exposed to a non-viremic partner.

## Results

### Patient groups

We studied 49 individuals from the San Francisco Positive Partners prospective couples cohort who were suppressing their virus while on HAART. The subjects were divided into two groups depending on the viral status of their partner, the viremic partner (VP) group, and the non-viremic partner (NVP) group. These groups did not differ in terms of the clinical parameters, such as age, time on therapy, CD4^+^ T cell count and viral loads (Table 1), or the level of sexual activity, as defined by the exposure score or the average number of insertive or receptive exposures (Table 2). However, there was a trend for higher levels of exposure in the subjects from the NVP group.

**Table 1:** Clinical parameters of each study group.

	Viremic Partner	Non-Viremic Partner	P-value
	(Mean±SEM)	(Mean±SEM)	(two-tailed)
Number (n = )	20	29	n/a
Participant's age (years)	42.20±1.76	41.76±1.75	0.86
Infection duration (days)	3934±535.40	4026±449.10	0.90
CD4 Count	503.1±60.95	559.2±56.45	0.51
Time on treatment (days)	2111±375.10	2594±373.20	0.38
Relationship Length (years)	4.05±1.13	3.22±0.90	0.56

**Table 2:** Sexual exposure and direction of exposure to a partner between the two groups.

	Viremic Partner Group	Non-Viremic Partner Group	P-value
	(Mean±SEM)	(Mean±SEM)	(two-tailed)
Exposure Score	0.07±0.02	0.11±0.02	0.23
Receptive Average	8.15±2.07	12.47±2.84	0.27
Insertive Average	7.05±1.80	12.71±2.82	0.13
Exposure Outside of Partnership	4.66±2.154	8.42±4.38	0.48

### Sexual exposure to a viremic partner boosts HIV-1 T cell specific responses

**Figure 1:**
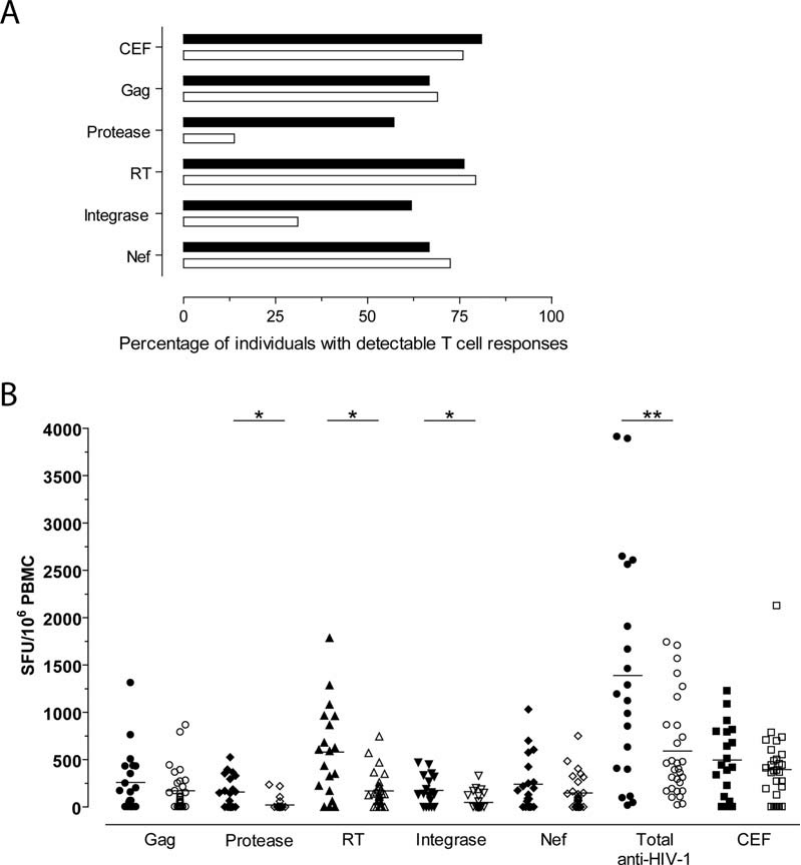
Responses to HIV-1 peptides and the CEF pool. A) Percentage of individuals making responses from either group: solid bars indicate the viremic partner group and open bars represent the non-viremic partner group. There was a significant difference in the number of individuals responding to Protease and Integrase peptide pools (p = 0.04 and p = 0.002 respectively), as determined by Fishers exact test. No difference was seen for any other stimuli. B) Magnitude of IFN-γ T cell responses to the HIV-1 or CEF peptide pools, and the total cumulative anti-HIV-1 response. Responses from individuals within the viremic partner group (closed symbols) and individuals within the non-viremic partner group (open symbols) showed significant differences for Protease, RT, Integrase and the total cumulative anti-HIV-1 response, *p<0.001 and **p = 0.0274 respectively. The median of each group is plotted as a horizontal line.

Analysis of T cell IFN-γ responses revealed significantly more individuals from the VP group made responses (as defined in the materials and methods section) to HIV-1 Protease and Integrase peptides, compared to individuals with the NVP group (Figure 1A). However, there was no difference in the percentage of individuals from each group making responses to HIV-1 Gag, Reverse Transcriptase (RT), Nef, or CEF. Further analysis of the individuals who made responses revealed that there was no significant difference in the magnitude of the responses made to either of these proteins (Figure 1B). However, Protease, RT, and Integrase (p<0.001) responses were significantly higher in the VP group compared to the NVP group. Overall, there was a trend towards a higher magnitude of response to HIV-1 Gag, Protease, Integrase and Nef by individuals with viremic partners (VP group). Moreover, cumulatively there was a significantly higher anti-HIV-1 T cell response made by the VP group (p = 0.0274).

### Frequency of continued sexual exposure determines the magnitude of the HIV-1-specific T cell response

**Figure 2:**
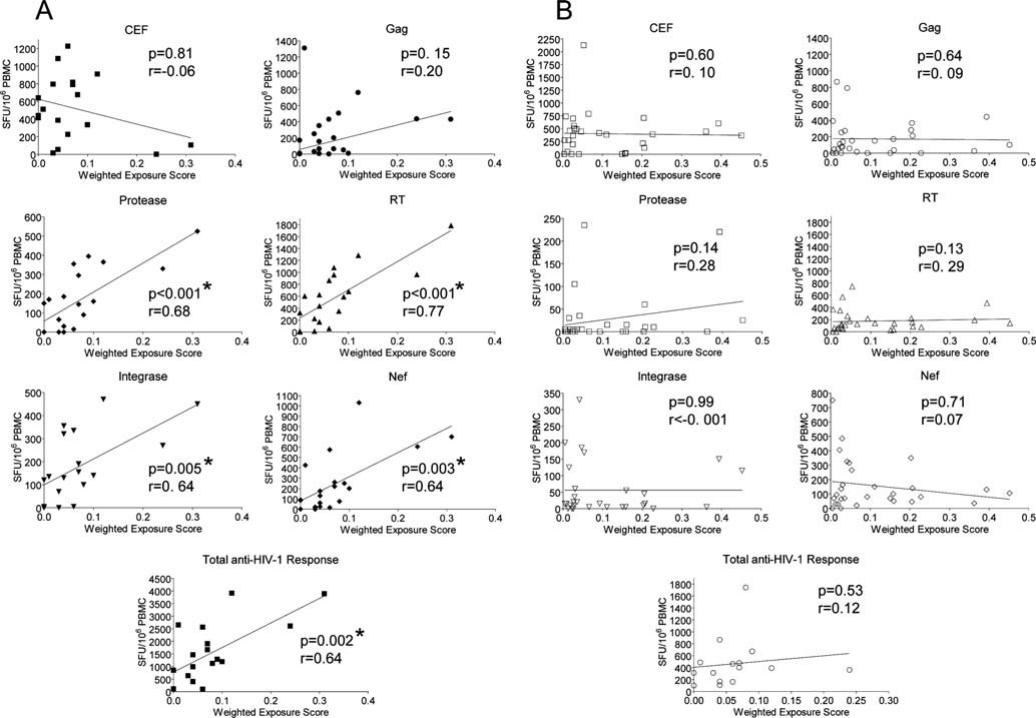
Correlations of exposure to a partner's HIV-1 and T cell IFN-γ response. IFN-γ T cell responses from the viremic partner (VP) group (A), or non-viremic partner (NVP) group (B), were plotted against the corresponding exposure scores against the peptide pools derived from CEF, or HIV-1 proteins Gag, Protease, RT, Integrase, and Nef. The cumulative anti-HIV-1 T cell IFN-γ response was also plotted. Significant correlations (denoted by *) were determined by two-tailed nonparametric spearmans correlation, spearman r (r), and linear regression analysis was plotted as a straight line.

We further explored the behavioral influence of exposure on the T cell response by utilizing data on the frequency of unprotected sexual intercourse in the partnerships. The exposure scores were plotted against the magnitudes of IFN-γ responses for individuals within each group (Figure 2). Individuals from the VP group showed strong correlations between their level of exposure and their T cell responses to peptide pools corresponding to HIV-1 Protease, RT, Integrase and Nef (Figure 2A). Gag and CEF peptide pools showed no significant correlation, although Gag responses did follow the same trend as the other HIV-1 proteins. In contrast, no correlations were found between the exposure scores and IFN-γ responses made by individuals whose partners were suppressing their virus (NVP group) (Figure 2B). Thus, not only is the level of exposure important in determining the magnitude of the T cell response, but critically it is exposure to a viremic partner that is associated with these responses.

### Receptive sexual exposure drives the T cell immune response

**Figure 3:**
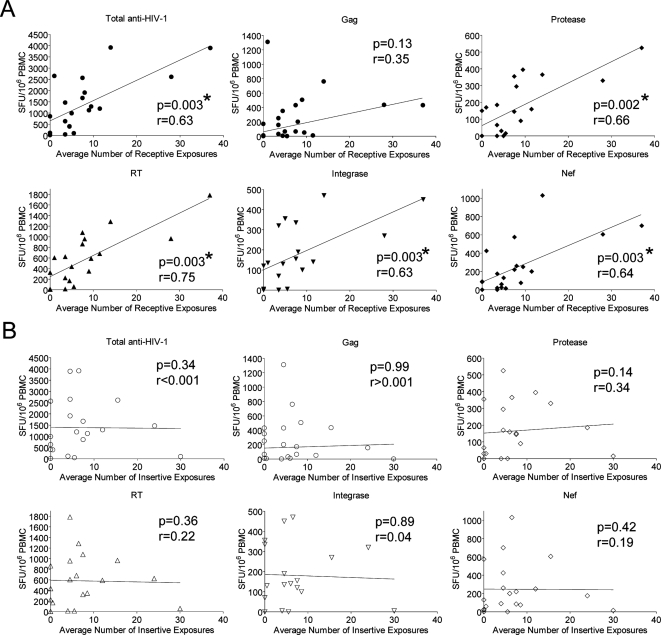
Direction of exposure and T cell IFN-γ response correlations. IFN-γ T cell responses from the viremic partner (VP) group were plotted against the corresponding average number of unprotected receptive (A) or insertive (B) anal exposures to a partner's HIV-1. Significant correlations (denoted by *) were determined by two-tailed nonparametric spearmans correlation, spearman r (r), and linear regression analysis was plotted as straight lines.

The type of exposure was further analyzed in the VP group by assessing the average number of unprotected receptive (Figure 3A) versus insertive (Figure 3B) anal intercourse episodes. All the individuals in this study engaged in both activities. Significant correlations were only seen between the IFN-γ responses and the number of receptive exposures. Again, responses against HIV-1 Protease, RT, Integrase, and Nef derived peptides correlated with the immune response. Gag followed the trend but did not reach significance, while CEF responses showed no correlation. There were no significant correlations between the average number of insertive exposures and IFN-γ responses towards HIV-1 Protease, RT, Integrase and Nef. As expected, data from the NVP group did not show a correlation between the IFN-γ responses and the direction of exposure (data not shown).

### Both CD4^+^ and CD8^+^ T cells made HIV-1 specific IFN-γ Responses

**Figure 4:**
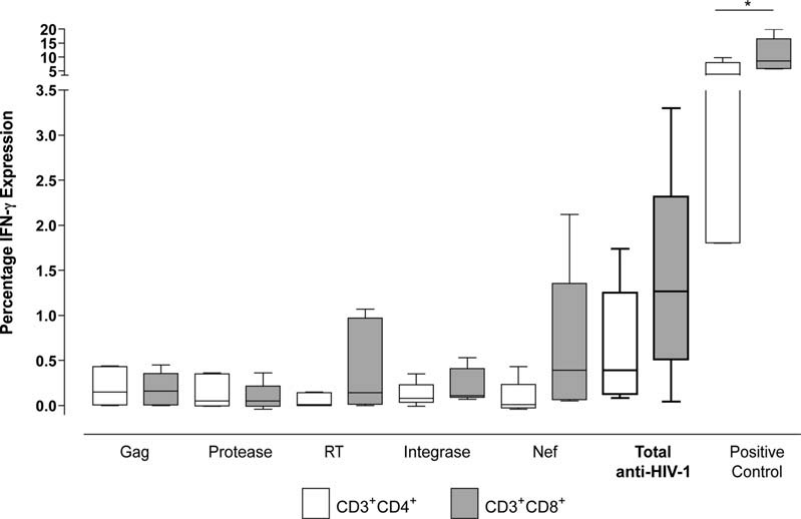
IFN-γ responses are derived from both CD4^+^ and CD8^+^ T cells. CD4^+^ (open boxes) and CD8^+^ (shaded boxes) T cell IFN-γ responses towards HIV-1 derived peptide pools Gag, Protease, RT, Integrase and Nef, plus the cumulative anti-HIV-1 response and a SEB positive control. No significant difference was observed between the two T cell subsets (n = 5).

To determine which T cell subset was responsible for the IFN-γ response detected in the ELISpot assay, individuals from the viremic partner group (n = 7) were screened by flow cytometry. Individuals were tested using the same 15mer peptide pools used in the ELISpot assay. Both CD4^+^ and CD8^+^ T cell subsets produced IFN-γ in response to all the HIV-1 peptide pools (Figure 4). However, no significant difference between the two subsets was seen, although there was a trend towards the CD8^+^ T cell subset.

### Exposure had no effect on poly-functionality or the level of total activation

**Figure 5:**
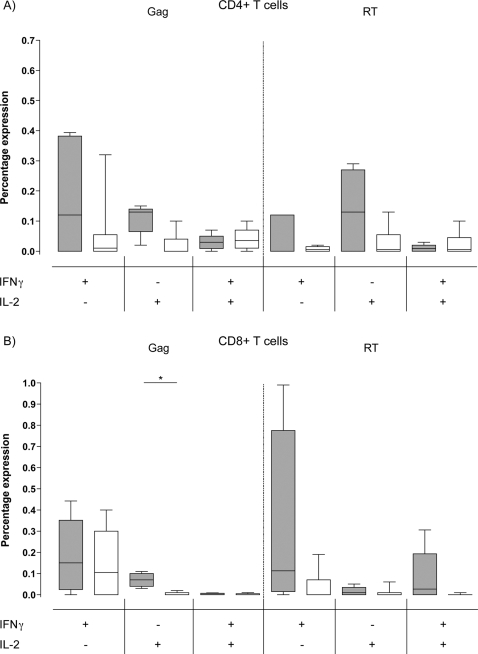
Exposure did not increase poly-functionality. The ability of CD8^+^ (A) and CD4^+^ (B) T cells to express IFN-γ and IL-2 was assessed in both the VP group (shaded boxes, n = 5) and NVP group (open boxes, n = 8). Poly-functionality was determined by the expression of both cytokines. Only the percentage of IL-2 expressing CD8^+^ T cells was significantly higher in the VP group (p = 0.0059), denoted by *.

In recent studies poly-functional T cells have been associated with slower disease progression [12]. PBMC from 8 individuals from the viremic partner group and 10 from the individuals from the non-viremic partner group were stimulated with 15mer peptide pools covering HIV-1 Gag and RT. CD4^+^ and CD8^+^ T cell expression of IFN-γ and IL-2 were measured by multi-parametric flow cytometry (Figure 5). No significant difference in the level of poly-functionality was seen between the two groups, although there was a trend for greater responses in the viremic partner group. IL-2 expression by CD8^+^ T cells was significantly higher (p = 0.0059) in the viremic partner group compared to the non-viremic partner group. In the positive control, SEB stimulation, revealed poly-functional T cells (data not shown).

**Figure 6:**
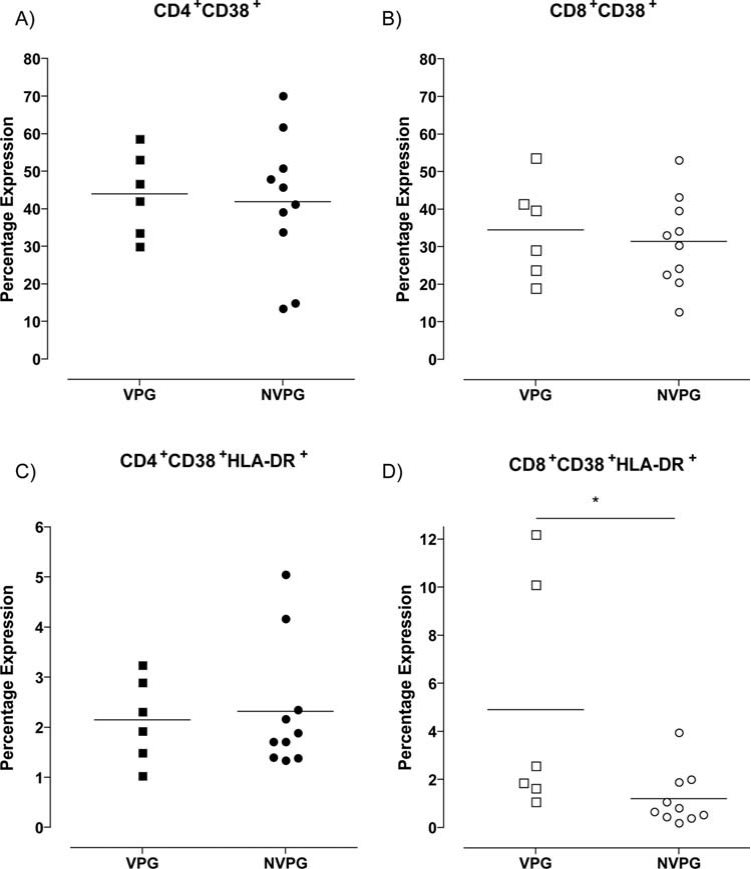
Total T cell activation was not influenced by exposure. The expression level of activation markers CD38 and HLA-DR were compared between VP group (squares, n = 6) and NVP group (circles, n = 10). The expression of CD38 on total CD4^+^ (A) and total CD8^+^ (B) T cells showed no significant difference. No significant difference was seen in CD4^+^ T cell co-expression of CD38 and HLA-DR (C). However, CD8^+^ T cells from the VP group co-expressed significantly higher of CD38 and HLA-DR compared to the NVP group (denoted by *) (D).

The total level of T cell activation was measured by CD38 and HLA-DR expression. No significant difference was seen in the level of expression of the activation marker CD38 on either CD4^+^ or CD8^+^ T cells (Figure 6 A and B). However, there was a significantly higher level of CD38^+^ and HLA-DR^+^ double positive CD8^+^ T cells in the viremic partner group (p = 0.0312), although this was not reflected in the CD4^+^ T cell subset (p = 0.5148) (Figure 6 C and D).

### Decreased exposure is mirrored by decreased T cell responses

**Figure 7:**
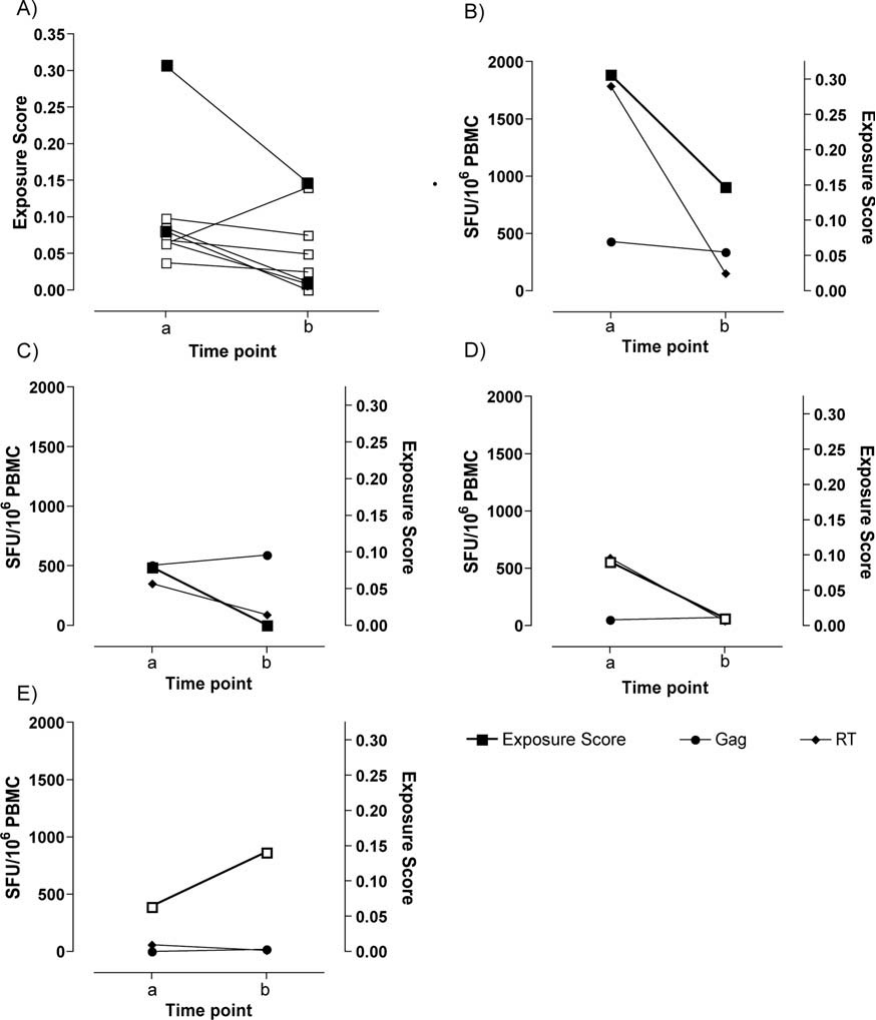
RT responses follow changes in exposure longitudinally. Exposure scores generated from questionnaires at the first time (time point 0) and 1 year later (time point 1) were compared for 7 individuals from the viremic partner group (A). Two individual's partners started anti-retroviral therapy between the time points and their viral loads were below the level of detection at the second time point (filled squares). ELISpot technology was employed to measure IFN-γ responses. Two individuals with aviremic partners at the second time point also showed a decrease in exposure to their partner (B and C), which was reflected in their anti-HIV-1 RT response. This was also seen in a third individual whose exposure dropped dramatically at the second time point (D). One individual showed an increase in their level of exposure, however, there was no responses was above background (<50 SFU) (E).

Seven individuals from the viremic partner group had PBMC samples available from a one year follow up time point. All the individuals continued to suppress their viral load below the level of detection (<50 copies/ml) and their CD4^+^ T cell count remained constant (Table 3). The exposure scores generated from the second time point showed little change in exposure levels in the majority of individuals (Figure 7A). However, two individuals from the viremic partner group had partners who started anti-retroviral therapy and were suppressing their viral loads at the second time point, one year after the first (Figure 7A filled squares). Both individuals also showed a drop in exposure, which was mirrored by a drop in the response to RT, although responses to Gag did not change (Figure 7B and C). A third individual whose exposure score dropped dramatically at the second time point also showed a drop in their RT response (Figure 7D). One individual had a modest increased the level of exposure to their partner compared to the pervious time point. However, no responses to either Gag or RT were detectable at either time point (Figure 7E).

**Table 3:** Clinical data of 8 individuals from VPG at time point 0 and 1 year later at time point 1.

Individual	Time point 0				Time point 1			
	Weighted Exposure Score	CD4 Count	Viral Load	Receiving therapy	Weighted Exposure Score	CD4 Count	Viral Load	Receiving therapy
1*	0.3100	636	<50	Yes	0.1469	637	<50	Yes
2	0.0900	654	<50	Yes	0.0112	462	<50	Yes
3	0.0700	598	<50	Yes	0.0246	575	<50	Yes
4	0.1000	452	<50	Yes	0.0747	432	<50	Yes
5	0.0800	294	<50	Yes	0.0080	434	<50	Yes
6*	0.0700	543	<50	Yes	0.0000	532	<50	Yes
7	0.0600	650	<50	Yes	0.0492	675	<50	Yes
8	0.0400	260	<50	Yes	0.1403	532	<50	Yes

*: indicates that the individuals partner started to receive anti-retroviral therapy after time point 0, and had a viral load below 50 copies per ml.

### No other clinical parameter influenced the T cell response

T cell responses can be influenced by a number of other factors in addition to direct viral antigen stimulation. In order to account for this, we analysed the responses measured in the VP group against a number of clinical parameters (Table 4). Only significant correlations could be found for the length of time the individuals were on therapy and responses to Protease and RT. However, this association was confounded by exposure. In a least squares linear regression model where time on treatment and exposure effects was independently controlled, time on treatment provided no additional explanatory power. In fact, the effect of treatment on both RT and Protease T cell responses completely washed out (r = 0.014, p = 0.94; and r = 0.14, p = 0.51 respectively), while the effect of exposure remained high and significant in the model overall (r^2^ =  0.56, p = 0.001; r^2^ = 0.551, p = 0.001 respectively). We found no correlation between any of the other parameters and the magnitude of the T cell IFN-γ responses.

**Table 4:** The influence of clinical parameters on T cell responses.

Viremic Partner Group	CEF	Gag	Protease	RT	Integrase	Nef	Total
**Age**							
Spearman r	−0.1570	0.2030	0.1160	0.2200	0.1410	0.0825	0.1740
P value (two-tailed)	0.5336	0.3910	0.6272	0.3650	0.5762	0.7295	0.4633
Significance	ns	ns	Ns	ns	ns	ns	ns
**Time on Therapy**							
Spearman r	−0.1020	0.3340	0.4850	0.4190	0.2350	0.3230	0.3550
P value (two-tailed)	0.6867	0.1506	0.0303	0.0738	0.3489	0.1642	0.1247
Significance	ns	ns	†	†	ns	ns	ns
**CD4 Count**							
Spearman r	−0.1520	−0.0497	0.3740	0.2900	0.1600	0.2950	0.2930
P value (two-tailed)	0.5479	0.8352	0.1045	0.2276	0.5256	0.2070	0.2096
Significance	ns	ns	Ns	ns	ns	ns	ns
**Partners Viral Load**							
Spearman r	0.2320	−0.2470	−0.1870	−0.0377	−0.0816	−0.0015	−0.0241
P value (two-tailed)	0.3538	0.2938	0.4292	0.8781	0.7475	0.9950	0.9198
Significance	ns	ns	Ns	ns	ns	ns	ns
**Infection duration**							
Spearman r	−0.1210	0.1650	0.2130	0.2610	0.1750	0.1200	0.1650
P value (two-tailed)	0.6332	0.4872	0.3675	0.2811	0.4884	0.6155	0.4858
Significance	ns	ns	Ns	ns	ns	ns	ns

*: indicates significant correlations.

**†:** denotes significant correlations that did not hold when tested by least squares linear regression.

ns = no significant correlation could be found.

### HIV-1 Super-infection?

**Figure 8:**
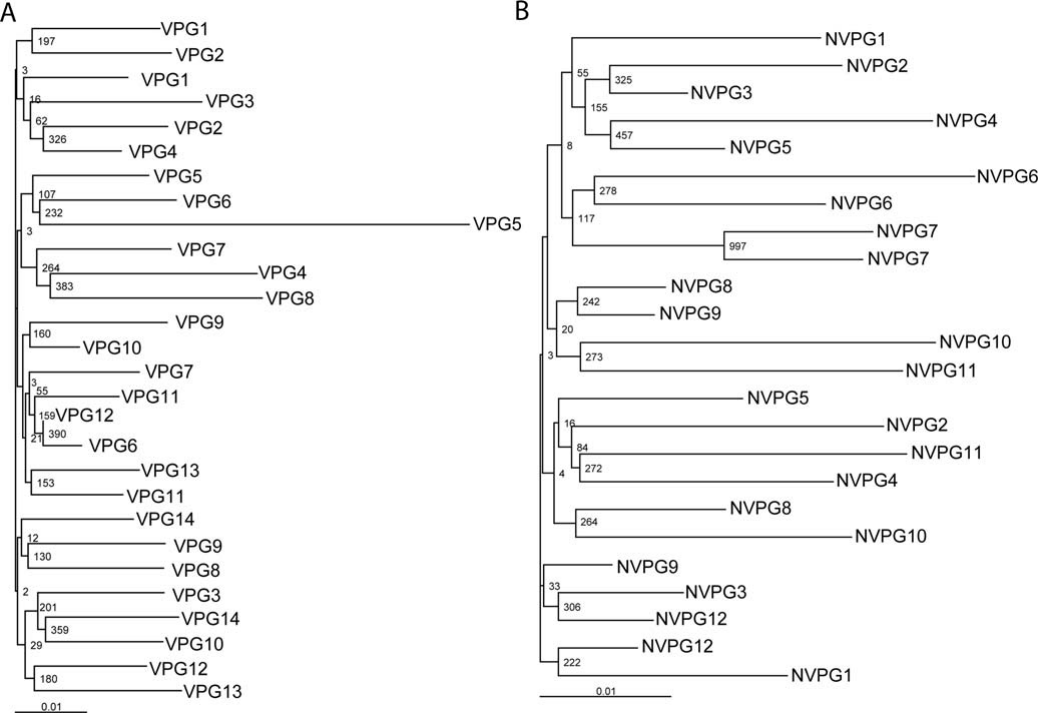
Phylogenetic analysis showed no evidence for systemic super-infection. No individuals studied in either the viremic partner group (A), or the non-viremic partner group (B) showed evidence of systemic super-infection by phylogenetic analysis. A unique partnership number indicates each partnership. No bootstrap values above 70% (indicated at each branch point) were generated between partner's viral sequences taken from the same time point, indicating their viruses were significantly divergent and no super-infection had occurred. * Known infecting partners.

The most likely mechanism, by which these responses are maintained or boosted in the individuals with viremic partners, is through the infection of host cells. Thus, potentially these individuals could be super-infected with their partner's HIV-1. To address this concern we used phylogenetical analysis to assess all the patients within this study at both Gag (data not shown) and Pol (Figure 8). Population analysis of the individual's cellular DNA and partner's plasma RNA revealed no evidence of systemic super-infection in any of the individuals studied within either group.

## Discussion

The consequence of continued exposure to HIV-1 in individuals already infected, to our knowledge has never been reported. This is becoming increasingly relevant with the advent of a growing trend for serosorting, the practice of seeking to only engage in unprotected sexual activities with partners who are of the same HIV-1 status [1],[2]. The study set out to measure the impact of continued exposure to HIV-1 on anti-HIV-1 T cell responses in individuals already infected.

Here, we have shown that HIV-1+ individuals who are regularly exposed to an HIV-1+ viremic long-term partner display greater HIV-1-specific CD4^+^ and CD8^+^ T cells responses, than infected subjects with aviremic partners. Furthermore, in these individuals the magnitude of the T cell IFN-γ response towards Pol and Nef significantly correlated with the level of exposure. Longitudinally, RT responses mirrored the level of exposures.

These observations are further strengthened by the fact that only the number of receptive events correlated with the T cell IFN-γ responses and not the number of insertive events. This is consistent with the observation that receptive intercourse represents a greater risk of acquiring HIV infection compared to insertive intercourse [13].

It is interesting to note that responses were predominantly directed towards HIV-1 Pol proteins rather than to HIV-1 Gag proteins. This is in agreement with Karlsson *et al*, who showed responses switch from predominantly Gag to Pol in individuals on antiretroviral therapy [14]. Moreover, it was the RT responses that mirrored decreases in exposure longitudinally, suggesting that RT-specific responses are more susceptible to antigen levels compared to Gag.

The responses observed reflect the engagement of host T cells with cells infected by exogenous HIV-1. These responses could be driven by three possible sources of viral antigen: via antigen presented on partner-derived cells within the seminal fluids, super-infection of host cells, or virion derived proteins.

The presentation of viral antigens on a partner's HLA can occur on partner-derived cells or cell-free HLA molecules within the seminal fluids [15],[16]. However, this is unlikely to be the sole mechanism for stimulating the host immune response, as this could only occur through HLA alleles that are shared by both partners. A more likely explanation involves the infection of host cells with a partner's virus.

Viral suppression within the peripheral blood by HAART has been shown not to be mirrored within the gut mucosal layer, where limited viral replication has been measured [17],[18]. Therefore, it is possible that the alleviation of drug pressure within the mucosal layer could allow a limited super-infection. Phylogenetic analysis of all the patients within this study revealed no evidence of systemic super-infection. However, this does not exclude limited or localized super-infections within the gut.

Free virus within the semen could provide enough antigenic stimuli to act as a “natural” immunogen. Proteins present in the viral particle have been shown *in vitro* to be sufficient to induce both cytotoxicity and IFN-γ secretion by CD8^+^ T cells, prior to viral replication within an infected CD4^+^ T cells [19],[20]. This suggests that a response could be stimulated *in vivo* in the absence of viral integration.

The ability of HIV-1 to induce an immune response, but remain undetectable at the peripheral level, has been shown in exposed uninfected individuals [21],[22],[23]. In particular this has been shown in health care workers who only received very limited HIV-1 exposure [24]. Furthermore, it is well established that discordant partners and sex workers, regularly exposed to HIV-1, can generate an anti-HIV-1 T cell response while remaining uninfected. However, it is noteworthy that this apparent protection can be lost after just temporarily ceasing exposure [25],[26],[27]. This suggests that HIV-1 can act as a potent immunogen, capable of generating immune responses either in the absence of infection or at levels below the current level of detection. Indeed, the site of inoculation appears to play a role too. A low dose of X4 SHIV_SF33A_ induces potent cellular immunity via the vaginal route of inoculation [28]. Furthermore, HIV-1 chronically infected individuals have been shown to exhibit robust multifunctional CD8^+^ T cell responses within the rectal tissue [29],[30]. Macaques initially infected with live attenuated SIV, show protection against subsequent intrarectal challenge with a more virulent strain [31],[32],[33],[34]. This protection from super-infection was thought to involve local CD8^+^ T cell immunity. However, in this study only limited differences between the level of poly-functionality and total activation were observed between the VP and NVP groups. This suggests that although exposure to exogenous HIV-1 can maintain responses, it cannot alter the functionality of the T cells, although further studies are needed.

The ability of exogenous HIV-1 to shape an immune response has important implications for HIV-1 infected individuals who choose to engage in unprotected sex with other HIV-1 infected individuals. The goal of therapeutic immunization strategies in HIV-1 infected people is to maintain strong anti-HIV-1 immune responses in individuals on HAART (reviewed in [35],[36]). Here, we have observed a “natural immunization” with live infectious virus delivered to the rectal mucosa. While the maintenance of an anti-HIV-1 response may be considered a positive outcome, individuals engaging in unprotected sexual intercourse with other HIV-1 infected individuals could be at risk of super-infection, which would be particularly detrimental if the super-infecting virus carried drug resistant mutations. Furthermore, these maintained responses remained limited in functionality, suggesting the “quality” of the responses was not improved.

Although none of the study participants showed evidence for super-infection at the systemic level (in which a new virus overgrows the resident strain), we cannot rule out potential compartmentalized super-infections within the rectal tissues, or super-infections that were cleared locally. Moreover, all of the study individuals were on successful HAART (VL<50 copies/ml), which could also limit infection with exogenous HIV-1.

We propose that HIV-1 exposure can lead to infection in susceptible individuals, but also act as a potent “natural” immunogen in individuals already infected. The mechanism/s behind the immunogenicity of exposure to a partner's HIV-1 remains unclear. However, we speculate that the maintenance of the anti-HIV-1-specific T cell response most likely reflects limited super-infection within the rectal tissues. However, catching a partner's HIV-1 in the act of super-infecting an exposed partner's tissues will be a technically challenging task.

This data also reveals a more general mechanism that occurs in infectious diseases; immune responses to chronic virus infections in a host are influenced not only by the chronic virus within the host, but also by exposure to exogenous virus from without.

## Materials and Methods

### Study design

We selected 49 individuals from the San Francisco Positive Partners prospective couples cohort study based on specific criteria: they had been on highly active antiretroviral therapy (HAART) for over 3 months and had viral loads under the limit of quantification on a sensitive assay (<50 copies/ml), they had a co-enrolled partner who was also HIV-1 positive and who was either viremic (the lowest viral load was >8000 copies/ml) or had a viral load <50 copies/ml. All subjects were men who have sex with men (MSM).

From these study subjects two groups were created based on the partner's viral load. The first group comprised of individuals with viremic partners, viremic partner (VP) group, and the second included subjects with virologically suppressed partners (<50 copies/ml), non-viremic partner (NVP) group. The two groups did not differ in terms of age, CD4 count, time on therapy, length of time infected, or time with partner (Table 1).

### Assessment of Exposure

Sexual exposure data were derived from self-administered questionnaires completed by both partners independently regarding sexual practices during the past three months. The instrumentation was based upon one of the few questionnaires ever developed to measure sexual behavior that has been extensively validated [37],[38]. It was adapted to the study of HIV-positive seroconcordant partnerships and extensively piloted and revised prior to the enrollment of the couples in this sub study. An exposure score was calculated from the number of times the subject reported they had had unprotected receptive anal intercourse with their partner, and the number of times their partner reported they had unprotected insertive anal intercourse with the subject. This gave an average receptive exposure score, which was multiplied by the associated risk of infection per receptive exposure for HIV-1 negative individuals through this type of exposure [13]. The insertive exposure score was calculated in a similar manner and multiplied by the associated risk of infection per exposure for unprotected insertive anal intercourse [13]. The sum of these averages gave a final exposure score. All immunological studies were performed blinded to the exposure data.

In addition to sexual exposure to an enrolled partner the same sexual exposure measures were asked of all other sexual partners in the past three months. There was no significant difference in exposure with non-enrolled partners between the VP and NVP groups (Table 2). Although the HIV-status of most non-enrolled partners was known, knowledge of treatment status was irregular, and neither self-reports nor laboratory values of viral load were available for these partners. Therefore, outside partnerships provided no additional data useful in this analysis.

### Study subjects

The study included 49 study subjects from the San Francisco Positive Partners prospective couples study. HIV-1-positive seroconcordant sexual partnerships were enrolled in this study if they reported unprotected intercourse. All subjects, in this sub-sample were MSM, who had been HIV-1 positive for over 2 years and were currently suppressing their virus, below 50 copies/ml, while on HAART.

### ELISPOT Analysis of T-cell responses

T cell responses were determined by IFN-γ ELIspot assay as previously described [39]. Overlapping peptides of 15–18 amino acids in length were employed, which encompassed HIV-1 consensus B (NIH) Gag, Protease, Reverse Transcription (RT), Integrase, and Nef. In addition, a non-HIV-1 viral peptide pool comprising of peptides from Cytomegalovirus, Epstein-Barr virus, and Influenza (CEF) was used as an additional control. Cryopreserved PBMCs were thawed and plated at 10^5^ cells were well, with a final peptide concentration of 1 µg/ml. All spot numbers were normalized to numbers of IFN-γ spot-forming units (SFU) per 10^6^ PBMCs. Spot values from medium control wells were subtracted to determine responses to each peptide. Responses were determined as either greater than two times background, or greater than 50 SFU/10^6^ PBMCs, which ever was the higher. All experiments were conducted blinded to the individuals exposure score.

### Cell staining and flow cytometric analysis

Cryopreserved PBMCs were thawed and washed with complete media. For functional assays PBMCs were incubated with 2 µg/ml peptide pools plus anti-CD28 and anti-CD49d (BD Biosciences, San Diego) at 1 µg/ml, as describe above and after 30 min Brefeldin A was added to the cultures. Cultures were left for a further 7 hours and 30 minutes at 37°C, washed and stained. Flourochrome conjugated antibodies directed against cellular molecules: CD3 (Beckman coulter), CD4, CD8, IFN-γ, and IL-2 (all BD Biosciences), were used to stain cells. In addition the activation markers CD38 and HLA-DR (both BD Biosciences), were also stained for *ex vivo* in conjunction with CD3 (Beckman coulter), CD4 and CD8 (both BD Biosciences). Positive controls were Staphylococcal enterotoxin B (SEB) (Sigma). Data was acquired with a LSRII (BD Biosciences), and analyzed using FlowJo software (TreeStar).

### Statistical analysis

Statistical significance and graphical presentations were completed using GraphPad Prism version 4.00 for Windows, GraphPad Software, San Diego California USA, www.graphpad.com, or Microsoft Excel version 2003, Microsoft Corporation. Differences in the proportion of individuals responding to the various antigens were analyzed by Fishers Exact test (two-tailed). Statistical analysis of the response magnitudes was completed using a Mann-Whitey test. Correlations were determined by two-tailed nonparametric Spearman correlation, Spearman r-values are also given. Linear regression analysis was plotted on each correlation as a straight line. Least squares linear regression models were executed using SPSS statistical package version 11.5.

### HIV-1 Sequencing

The sequence of HIV-1 reverse transcriptase and protease was performed using the TRUGENE HIV-1 RNA genotyping kit and OpenGene system software for sequence analysis (Siemens Medical Solutions Diagnostics) [40],[41]. Mixtures are designated with standard ambiguity codes when representing 30% or greater minor variant at any particular base. Viral RNA from blood plasma was extracted using the Qia-Amp viral RNA kit (Qiagen), reverse transcribed, amplified and sequenced in subjects with sufficient viremia (>100 copies/mL). Proviral DNA from PBMC was amplified after extraction using the DNeasy tissue kit (Qiagen) and sequenced in samples from subjects with low (<100 copies/mL) or undetectable (<50 copies/mL) plasma viremia. Codons 1–99 of protease and 40 through 247 of reverse transcriptase were sequenced and analyzed in all samples.

### Phylogenetic Analysis

HIV-1 sequences were obtained from the HIV-1 Pol region and sequenced. All sequences were assembled using BioEdit v.7.0.4.1 and aligned with the Clustal X v1.83 sequence alignment tool. Phylogenetic analysis was done using neighbor-joining trees with bootstraps generated using Clustal X. We used 1000 random samples of sites from the alignment, drawing 1000 trees (1 from each sample) and counted how many times each grouping from the original tree occurs in the sample trees.
